# Exploring the dynamics of self-efficacy, resilience, and self-management on quality of life in type 2 diabetes patients: A moderated mediation approach from a positive psychology perspective

**DOI:** 10.1371/journal.pone.0317753

**Published:** 2025-01-24

**Authors:** Zheng Ting, Wang Huicai, Zakeer Kudelati, Ge Yongkang, Ayimire Alimu, Zhang Xiaotian, Qu Xingge, Li Tong

**Affiliations:** 1 Nursing School, Xinjiang Medical University, Urumqi, China; 2 Department of Undergraduate Educational Management, The First Affiliated Hospital of Xinjiang Medical University, Urumqi, China; 3 College of Health Management, Xinjiang Medical University, Urumqi, China; 4 The First Affiliated Hospital of Xinjiang Medical University, Urumqi, China; University of Illinois Urbana-Champaign, UNITED STATES OF AMERICA

## Abstract

**Objectives:**

Type 2 diabetes mellitus (T2DM) significantly deteriorates patients’ quality of life (QOL). This study examined the dynamic interplay of factors that influence QOL in patients with T2DM, utilizing concepts from positive psychology and intrinsic mechanisms, to lay the groundwork for improving patient outcomes. Improving self-management behaviors is essential for effective disease management.

**Methods:**

Using a cross-sectional design, this study incorporated 408 patients with T2DM from the endocrinology department of a public hospital in Urumqi, who were selected through convenience sampling from December 29, 2023 to June 30, 2024. Data collection tools included the General Data Questionnaire, Summary of Diabetes Self-Care Activities, Self-Efficacy for Diabetes Questionnaire, Connor-Davidson Resilience Scale, and Diabetes-Specific Quality of Life Scale. Structural equation modeling and Model 15 of Hayes’ SPSS-Process program facilitated the moderated mediation analysis.

**Results:**

The findings demonstrated that self-efficacy significantly enhanced the QOL (β = -0.8557, *p* < 0.01), with resilience serving as a partial mediator, accounting for 43.1% of this effect. Interactions between self-efficacy and self-management, and resilience and self-management, were also significant predictors of QOL (β = -0.0751, *p* < 0.01 and β = -0.0073, *p* < 0.05, respectively).

**Conclusion:**

These findings introduce a novel theoretical framework for T2DM from the perspective of positive psychology, which will be beneficial for intervention development. This study underscores the importance of promoting diabetes self-management as an effective strategy to enhance QOL. Additionally, healthcare providers must focus on fostering patients’ positive psychological traits and reliable self-management behaviors.

## Introduction

Type 2 diabetes mellitus (T2DM) is a metabolic disorder characterized by chronic hyperglycemia due to inadequate insulin secretion or insulin resistance. It poses a significant public health challenge and threatens societal well-being [1,2]. Diabetes is one of the fastest-growing global health emergencies of the 21st century, with 537 million people aged 20–79 years in 2021, possibly increasing to 743 million by 2045 [3]. The rising burden of T2DM is a major healthcare concern worldwide, with a considerable impact on human life and health expenditures [4]. Quality of life (QOL), which encompasses physical, psychological, and social facets, is crucial for gauging an individual’s overall health status. With a shift toward a bio-psycho-social medical model, QOL has become a key indicator for assessing patients’ health and psychological well-being, effectively predicting their overall health status [5]. The evaluation is based on the concept of comprehensive assessment of life values in patients. QOL is an important health outcome representing the ultimate goal of all health interventions [6]. T2DM significantly affects the patients’ QOL, affecting their physical health, psychological state, and social functioning [7]. Poor glycemic control over the long term can lead to complications, such as cardiovascular and cerebrovascular diseases, diabetic nephropathy, and diabetic retinopathy, further deteriorating the patients’ QOL [8]. Emerging evidence suggests that diabetes-related stigma and the associated negative emotions may impair the QOL of patients with T2DM [9]. Furthermore, people with diabetes have a higher risk of depression and diabetes-related distress, and poorer overall QOL [10].

Consequently, diabetes presents a substantial adverse stimulus for patients, necessitating effective emotional self-management to derive a positive meaning from their illness, thereby enhancing their QOL. Maintaining the QOL of patients with diabetes is a decisive outcome variable in the treatment [11]. It should be used as an essential quality indicator to evaluate the efficacy and effectiveness of treatment measures. Positive psychology emphasizes the importance of patients’ roles in psychological adjustment and explores and fosters positive psychological qualities to enhance adaptability, disease management, and psychosocial health, ultimately improving QOL[12]. Positive psychology offers a novel perspective on psychological nursing. To date, the relevant factors and mechanisms influencing the QOL of patients with T2DM from the perspective of positive psychology have not been clarified and require further exploration. Psychological resilience and self-efficacy, key attributes of positive psychology [13–15], focus on harnessing patients’ potential positive emotions and transforming them into constructive forces. This study investigated the factors influencing QOL in patients with T2DM through the lens of positive psychology, thereby providing a theoretical foundation for clinical nursing interventions.

Self-efficacy refers to an individual’s confidence in executing specific health-related tasks or behaviors under pressure or challenge. It directly influences cognitive and behavioral patterns and serves as a vital psychosocial determinant of diabetes self-management and health outcomes. Bandura’s self-efficacy theory posits that enhancing self-efficacy can bolster patients’ confidence in confronting the disease and encouraging proactive help-seeking behaviors, thereby alleviating stress and psychological distress and enhancing psychological resilience [16]. Psychological resilience refers to the capacity to effectively face and adapt to adversity, trauma, threats, or significant stressors, demonstrating an individual’s ability to recover from challenges[17]. Resilience is vital in personal development, as it aids in navigating life difficulties, improving life satisfaction, and enhancing overall happiness. Resilience robustly predicts life satisfaction, which increases with higher resilience levels [18]. Previous studies show that resilience is intrinsically linked to QOL, with higher resilience significantly enhancing it [19]. Patients with T2DM who present with elevated self-efficacy exhibit greater cognitive appreciation and overall life evaluation, stemming from disease management, experience more positive emotions, and possess heightened confidence in emotion regulation, which may better counterbalance the negative emotional impact of the disease. Consequently, these patients have a higher QOL. Drawing on this rationale, we posit the following Hypothesis 1: Higher levels of self-efficacy in individuals with T2DM are associated with enhanced QOL, with resilience playing a partial mediating role in the correlation between self-efficacy and QOL.

**Fig 1:**
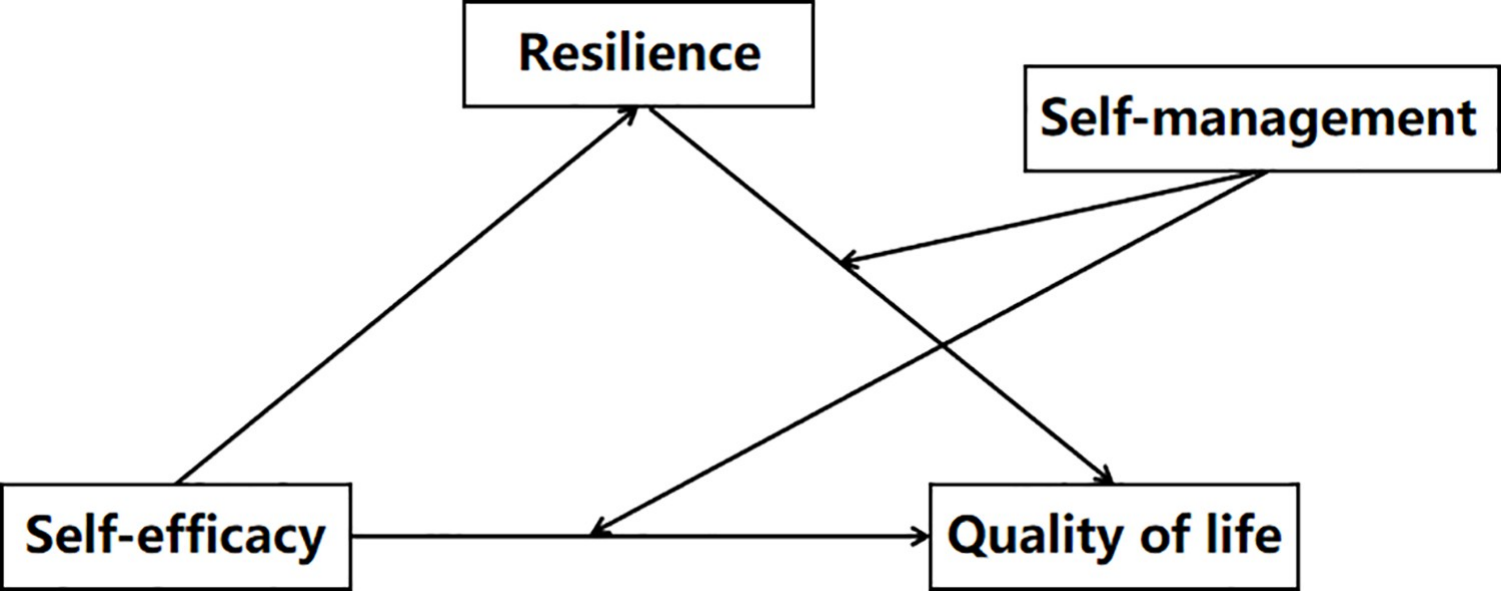
The proposed moderated mediation model.

The prognosis and QOL of patients with T2DM are intrinsically linked to their self-management levels. Effective self-management behaviors can regulate blood glucose levels, reduce hospitalization, mitigate the onset or progression of complications, and enhance health outcomes and QOL [20]. Effective disease control in patients with T2DM depends on robust self-management, which is a prerequisite for high self-efficacy [21]. Previous studies have shown that in goal-oriented behaviors, the feeling component of emotions contributes to the evolution of consciousness, cognition, and action processes, resulting in behaviors [10]. We infer that a dynamic interaction exists between self-efficacy and individual behavior; self-efficacy plays a constructive role in shaping behavior, with healthy lifestyle practices fostering a strong sense of self-efficacy, ultimately leading to a higher QOL. Studies show that diabetes-related distress directly or indirectly affects patients’ resilience [17]. Resilience development involves nurturing tenacity, adjusting adaptability, and transforming potentially negative psychology into positive psychology, thereby promoting resilience [22]. Patients with proficient self-management practice experience may be better at reducing diabetes-related distress, gradually becoming more resilient and optimistic, resulting in higher QOL. Accordingly, Hypothesis 2 posits that self-management may moderate the association between self-efficacy and QOL, as well as between resilience and QOL. Building on these hypotheses, a moderated mediation model is proposed (Fig 1). Through hypothesis testing and model validation, this study delves delved into the mechanisms and pathways through which self-efficacy, resilience, and QOL influence individuals with T2DM from the perspective of positive psychology. Additionally, we examined the modulatory role of self-management within this pathway. The subsequent sections present the findings of this study.

## Methods

### Design and participants

This cross-sectional study included 408 patients with T2DM from the endocrinology department of a tertiary grade-A hospital in Urumqi, West China, who were selected via convenience sampling. The inclusion criteria were: (1) Diabetes diagnosis based on the American Diabetes Association: 2-h PG ≥ 200 mg/dL (11.1 mmol/L) during OGTT [23]; (2) Diabetes history of ≥6 months; (3) Age ≥18 years; (4) Normal cognition, with the ability to understand and correctly respond to the questionnaire; and (5) Informed consent and willingness to complete the survey. Exclusion criteria were as follows: (1) Patients with mental health disorders or brain tumors who have severe communication and cognitive impairments; (2) Acute diabetic complications, such as diabetic ketoacidosis, or in a comatose state and unable to perform self-care tasks. That is, the complications prevent them from communicating properly; (3) Presence of other serious medical conditions, such as a serious cardiovascular disease, serious infectious diseases, cancer, visual and hearing impairments due to diabetes complications, etc., which make the patient weak and unable to take the questionnaire; (4) Patients with Type 1 diabetes or other specific types of diabetes.This study used structural equation modeling to test for moderated mediation effects, and a minimum sample of 290 participants was considered appropriate [17]. According to previous research, a sample size of 10–15 times the number of variables was required [7]. A total of 34 variables were included in this study. The final sample size was set at 408–612, accounting for 20% of non-responders. This study was approved by the Medical Ethics Committee of the First Affiliated Hospital of Xinjiang Medical University (K202312-18).

### Data collection

From December 29, 2023, to June 30, 2024, a questionnaire survey was conducted in the endocrinology department of a tertiary grade-A hospital in Urumqi by a team comprising of one graduate student and five undergraduate students. They were given uniform guidance and received standardized training, such as questionnaire distribution, collection skills, and communication skills before the survey. All participants who met the inclusion criteria were provided with standardized instructions and signed a consent form. Questionnaires were administered and collected on the spot, and each questionnaire was checked item by item during the collection process to ensure the authenticity and integrity of the data. The completed questionnaires were collected immediately after completion of the study. Ultimately, 420 individuals with T2DM were enrolled in this survey, of which 408 with complete data were eligible for data analysis, resulting in a response rate of 97.1%. The detailed contents of the questionnaires are as follows:

### Survey instruments

The questionnaire started with basic demographic information, such as gender, age, education level, and marital status. It also included specific queries related to diabetes management and health status, such as body mass index (BMI), presence of comorbidities, complications arising from diabetes, duration of diabetes in years, and details of the use of medications related to diabetes management.

The Summary of Diabetes Self-Care Activities (SDSCA) scale, initially developed by Toobert in 2000 [24], is a concise tool for assessing self-care behaviors in individuals with diabetes. The Chinese version of the SDSCA comprises 11 items divided into five subscales, evaluating exercise (2 items), diet control (4 items), medication (1 item), blood glucose monitoring (2 items), and foot care (2 items) [25]. It assesses the frequency of specific self-care activities over the previous week, with each item scored on a scale ranging from 0 (no days) to 7 (every day). The total score ranged from 0 to 77, with higher scores reflecting more effective self-management. In this study, the scale showed robust internal consistency,with a Cronbach’s α of 0.83.

The Self-Efficacy for Diabetes (SED) scale comprises eight items designed to measure self-efficacy across several domains critical for diabetes management, including dietary habits, physical activity, and overall disease management [26]. Each item on the scale is scored from 1 to 10, where 1 represents no confidence and 10 represents complete confidence in managing the respective aspects of diabetes care. The overall self-efficacy score is derived by calculating the average of the eight items, with higher scores indicating greater self-efficacy. In the current study, the reliability of this scale was confirmed by a Cronbach’s α of 0.87.

The Chinese version of the Connor-Davidson Resilience Scale (CD-RISC) was employed to measure participants’ ability to cope with adversity [27]. The scale comprises 25 items, each scored on a 5-point Likert scale ranging from 0 (never) to 4 (always). The cumulative score could reach 100, with higher scores indicating stronger resilience. The scale explores three key aspects–resilience, self-improvement, and optimism–with higher scores reflecting more robust psychological resilience. The reliability of the scale in this research was confirmed with a Cronbach’s α of 0.94.

The Chinese version of the Diabetes Specific Quality of Life Scale (DSQL), compiled by Liao Zhihong et al. [28], is structured into four dimensions: physiological function (12 items), psychological/spiritual (8 items), social relationships (4 items), and treatment (3 items), totaling 27 items. Each item is rated on a scale from 1 to 5, corresponding to “none at all,” “occasionally,” “sometimes (about half of the time),” “often,” and “always.” The sum of the item scores represents the total score, with higher scores indicating lower QOL. A total score of ≥80 suggests a low QOL, scores between 40 and 80 indicate a medium QOL, and scores ≤ 40 reflect high QOL. In this study, the scale demonstrated an overall Cronbach’s α of 0.91.

### Statistical analysis

Data analysis was performed using SPSS 26.0, with double data entries to ensure accuracy. Qualitative data were described as case numbers and percentages (%), while quantitative data conforming to a normal distribution were summarized as mean ± standard deviation (x ± s). Variable relationships were examined using Spearman’s correlation analysis. The structural equation modeling (SEM) approach was applied using IBM SPSS AMOS version 26 to explore the mediating effect of resilience on self-efficacy and QOL. The model’s fit was examined utilizing several criteria: χ^2^/df < 5, goodness of fit index (GFI) > 0.90, Tucker-Lewis index (TLI) > 0.90, comparative fit index (CFI) > 0.90, root mean square error of approximation (RMSEA) < 0.080, and standardized root mean square residual (SRMR) < 0.080, ensuring the model adequately matched the observed data [29]. Control variables included sociodemographic and disease-related characteristics such as age, gender, education level, and duration of diabetes. A moderated mediation analysis, designated as Hypothesis 2, was tested using Hayes’s PROCESS 4.1 for SPSS v. 26 (Model 15). This analysis included 95% bootstrap confidence intervals (CI) derived from 5,000 bootstrapped samples, with CIs excluding 0 denoting significant effects. Statistical significance was set at *p* < 0.05 (two-tailed). Harman’s single-factor analysis assessed the common method bias, revealing that 19 factors with eigenvalues > 1 explained 65.20% of the variance, with the first factor explaining 15.85%, which was below the 40% threshold. Therefore, no significant methodological biases were observed.

## Results

### Sample characteristics

The mean age of participants in the present study was 57.76 ± 10.79 years, with 42.4% being over 60 years of age. The sample included 42.2% females and 88.7% were married. Education levels varied, with 42.4% of the participants not holding a high school diploma. Regarding the duration of T2DM, 47.8% had been diagnosed for less than 10 years, whereas 65.2% were deemed overweight based on their BMI. A significant majority (96.1%) of the patients undergo oral insulin or insulin treatment. Furthermore, 41.2% reported a per capita monthly household income exceeding 5000 yuan, 85.8% had basic medical insurance for urban workers, and 81.6% resided in urban areas. A family history of diabetes was noted in 54.4% of the patients, 48.5% reported having more than one diabetic complication, and 60.3% had additional comorbidities. Notably, 82.4% of participants actively sought information on diabetes treatment (Table 1).

**Table 1:** Participant characteristics (N = 408).

Characteristic	Mean±SD	Frequency	Percentage
**Age (years)**	57.76±10.79		
<40		32	7.84
41–50		62	15.20
51–60		141	34.56
61–70		127	31.13
71–80		46	11.27
**Gender**			
Female		172	42.20
Male		236	57.80
**Years of illness**			
0–5		98	24.02
5–10		97	23.77
11–15		94	23.04
16–20		66	16.18
>20		53	12.99
**BMI (kg/m**^**2**^)			
BMI<18.5		6	1.47
18.5≤BMI≤23.9		136	33.33
24≤BMI≤27.9		185	45.34
BMI≥28		81	19.85
**Education levels**			
Illiterate		75	18.38
Less than high school diploma		193	47.3
High school diploma		83	20.34
Academic		57	13.97
**Marital status**			
Single		46	11.27
Married		362	88.73
**Per capita monthly household income (CNY)**			
<3000		64	15.69
3000–5000		176	43.14
5000–10000		127	31.13
>10000		41	10.05
**Payment of medical expenses**			
Basic medical insurance system for urban works		350	85.78
Rural Cooperative medical insurance		46	11.27
Others		12	2.95
**Address**			
Rural area		75	18.38
Urban area		333	81.62
**Family history of the disease**			
No		186	45.59
Yes		222	54.41
**Complications**			
No		210	51.47
1 Complication		77	18.87
2 Complications		89	21.81
3 Complications		25	6.13
4 or 5 Complications		7	1.72
**Comorbidity**			
No		162	39.71
1 Comorbidity		97	23.77
2 Comorbidity		91	22.30
3 Comorbidity		43	10.54
4 or 5 Comorbidity		15	3.68
**Diabetes-Related Medication Use**			3.68
None		16	3.92
Oral Meds		125	30.64
Insulin		47	11.52
Both		220	53.92
**Actively sought out information on diabetes treatment**			
Yes		336	82.35
No		72	17.65

### Correlational findings

Correlation analysis revealed that self-efficacy exhibited a positive and significant association with resilience (r = 0.347, *p* < 0.01) and self-management (r = 0.364, *p* < 0.01). Furthermore, there was a significant negative correlation between self-efficacy and QOL scores (r = -0.179, *p* < 0.01). Resilience also demonstrated a significant negative correlation with QOL scores (r = -0.276, *p* < 0.01) (Table 2).

**Table 2:** Correlations, means and standards deviations of study variables.

	Self-efficacy	Resilience	Self-management	Quality of life
Self-efficacy	1			
Resilience	0.347**	1		
Self-management	0.364**	0.072	1	
Quality of life	-0.179**	-0.276**	0.100*	1

** *p* < 0.01

* *p* < 0.05.

### Mediation analyses

**Fig 2:**
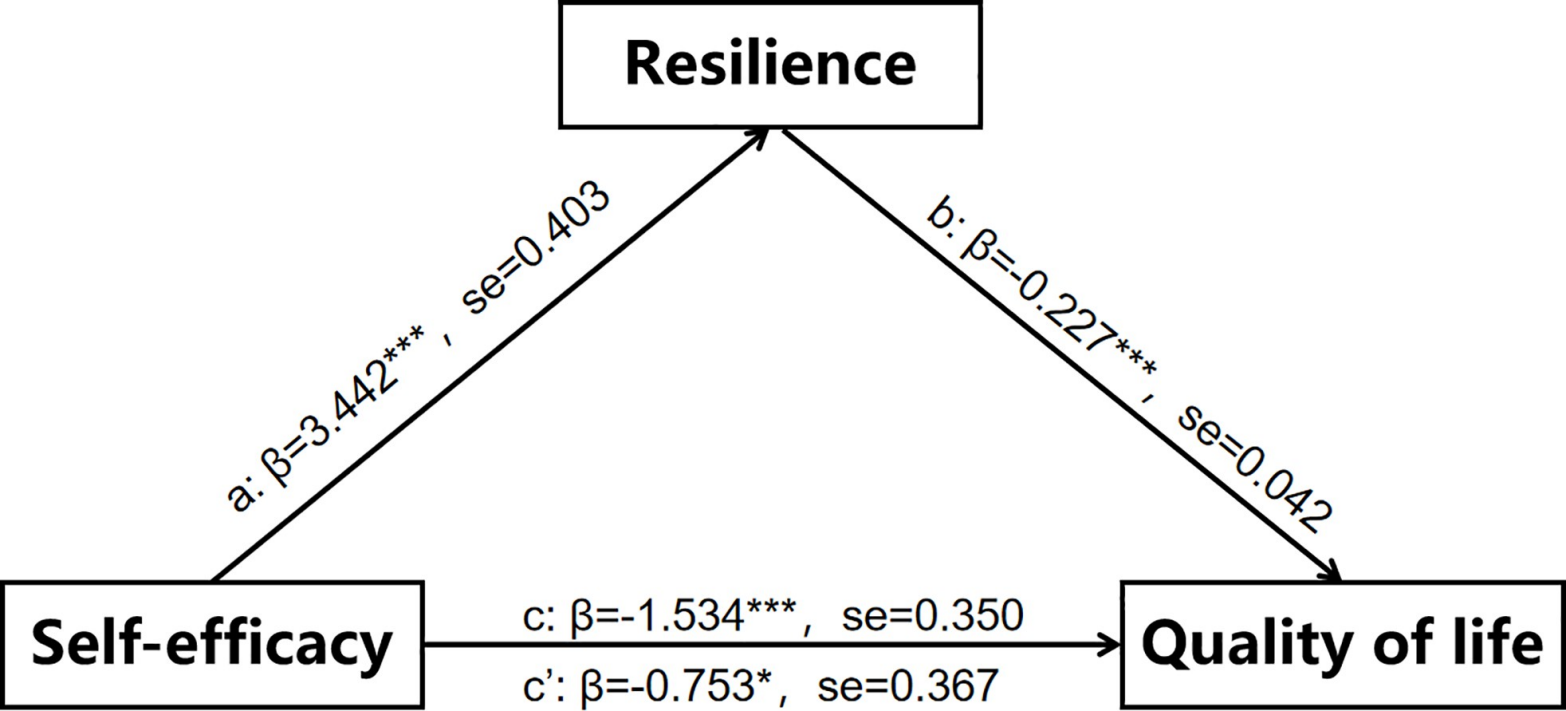
Hypothesized mediated model. Path c: Total direct effect, path c’: Direct effect. a: Effects of Self-efficacy on the mediator (Resilience); b: Effects of the mediator on the Quality of life. Using structural equation model (SEM).

Prior to data analysis, the dataset was thoroughly checked for missing values, outliers, and normal distributions. Confirmatory factor analysis was employed to validate the measurement model and assess the correlations between the observed variables and latent constructs. All factor loadings were greater than 0.50, indicating satisfactory values. The model’s goodness-of-fit was confirmed by modified indices: χ^2^/df = 1.646 (29.627/18 < 3), GFI = 0.984, AGFI = 0.960, CFI = 0.992, TLI = 0.984, NFI = 0.980, and RMSEA = 0.04, all suggesting a strong fit with the observed data. ***As depicted in Table 3 and Fig 2, the total effect of self-efficacy on QOL (path c) was significant (t = -4.381, β = -1.534, p < 0.001)***. Bias-corrected bootstrap CI based on 5,000 samples were used to test direct and indirect effects. The absence of 0 in the CI confirmed the significance of this correlation. ***The direct effect of self-efficacy on QOL (path c’) was also significant (β = -0*.*753*, *95% CI = [-1*.*476*, *-0*.*030]*, *p < 0*.*05)*. *Furthermore*, *the indirect effect of self-efficacy on QOL*, *mediated by resilience*, *was significant (β = -0*.*781*, *95% CI = [-1*.*283*, *-0*.*410]*, *p < 0*.*001)*, *indicating a partially mediating effect of resilience***.

**Table 3:** Total, direct, and indirect effects of each path in this model using structural equation model.

path	β	SE	BC95%CI	
			Lower	Upper
**Total effect**				
Self-efficacy→Quality of life	-1.534***	0.350	-2.420	-0.753
**Direct effect**				
Self-efficacy→Resilience	3.442***	0.403	2.523	4.414
Self-efficacy→Quality of life	-0.753*	0.367	-1.476	-0.031
**Indirect effect**				
Self-efficacy→Resilience→Quality of life	-0.781***	0.226	-1.283	-0.41

*N* = 408.

* p < 0.05

** p < 0.01

*** p < 0.001.

### Results of moderated mediation analysis

**Fig 3:**
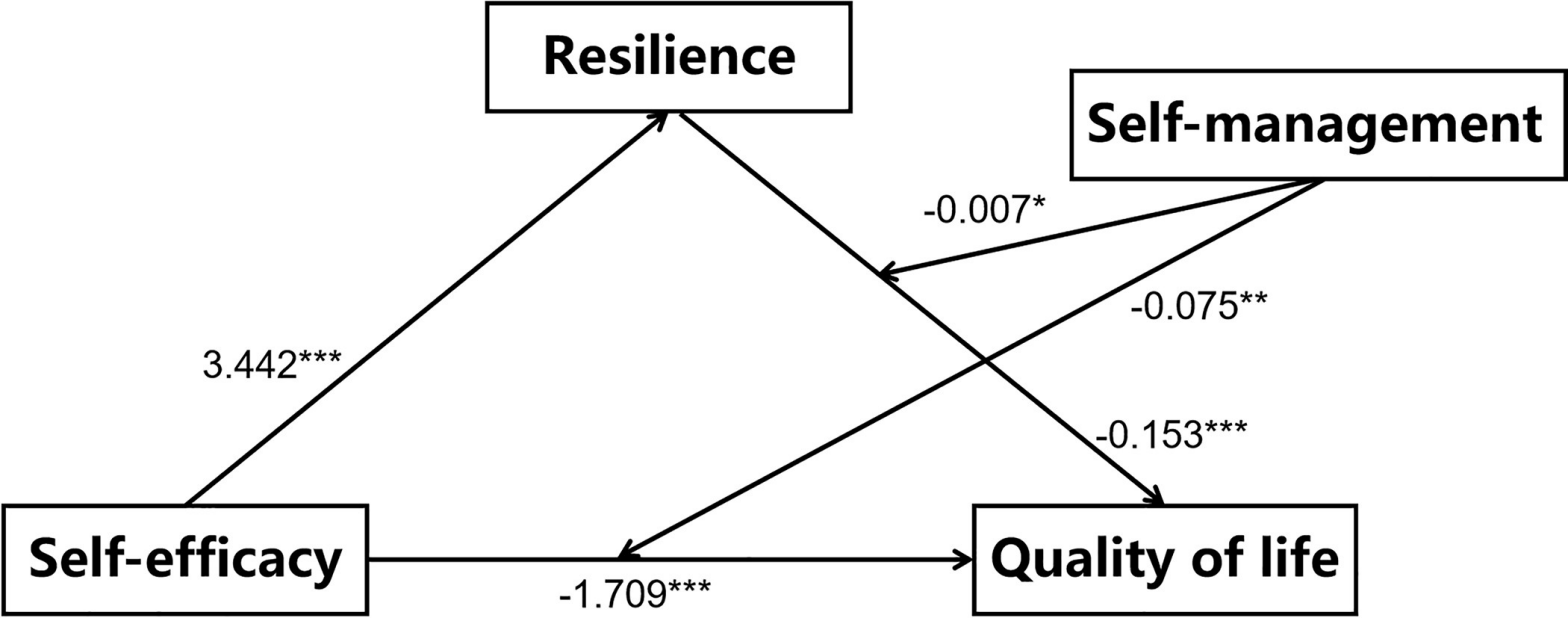
The moderated mediation model; *p < 0.05, **p < 0.01, ***p < 0.001.

As illustrated in Table 4 and Fig 3, Model 1 demonstrated that self-efficacy significantly impacted QOL (β = -1.709, SE = 0.374, 95% CI = [-2.445, -0.974], *p* < 0.001). In addition, resilience significantly affects QOL (β = -0.153, SE = 0.038, 95% CI = [-0.229, -0.078], *p* < 0.001). ***The interaction between self-efficacy and self-management shows a significant negative association with QOL scores (β = -0*.*075*, *SE = 0*.*024*, *95% CI = [-0*.*122*, *-0*.*028]*, *p < 0*.*01)*. *Similarly*, *the interaction between resilience and self-management is significantly negatively correlated with QOL scores (β = -0*.*007*, *SE = 0*.*003*, *95% CI = [-0*.*013*, *-0*.*002]*, *p < 0*.*05)***. These findings indicate that self-management moderates the relationships between self-efficacy and QOL as well as resilience and QOL, thus supporting Hypothesis 2.

**Table 4:** Results of the moderated mediation model analysis.

Variable	Model 1 Quality of life				
	β	SE	t	P	95%CI
Gender	0.9282	1.1630	0.7981	0.4253	-1.3584, 3.2147
Diabetes duration	0.2454	0.4441	0.5527	0.5808	-0.6276, 1.1185
Age	0.6751	0.5970	1.1308	0.2588	-0.4987, 1.8489
Education levels	0.1304	0.4893	0.2665	0.7900	-0.8317, 1.0924
self-efficacy	-1.7091	0.3741	-4.5679	0.0000	-2.4447, -0.9735
resilience	-0.1532	0.0384	-3.9860	0.0001	-0.2287, -0.0776
self-management	0.1209	0.0450	2.6847	0.0076	0.0324, 0.2094
self-management×self-efficacy	-0.0751	0.0240	-3.1330	0.0019	-0.1223, -0.0280
self-management×resilience	-0.0073	0.0029	-2.5405	0.0115	-0.0129, -0.0016
R^2^	0.3123				
F	11.0988				

Using Hayes’ PROCESS (Model 15) in the SPSS.

BMI Body mass index, CI Confidence interval.

N = 408.

**Fig 4:**
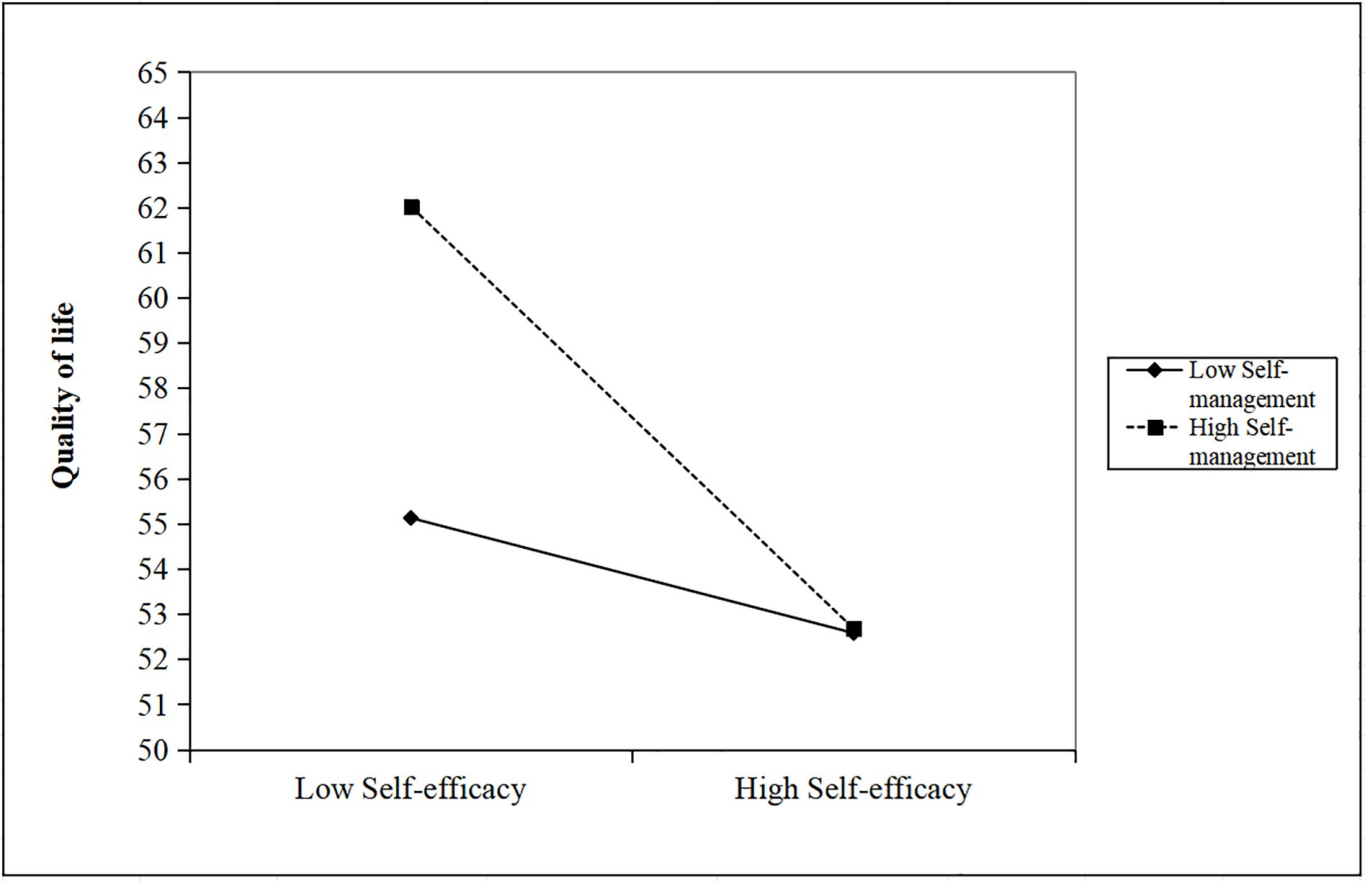
James Gaskin plot showing the interaction effect of self-efficacy and self-management on quality of life.

**Fig 5:**
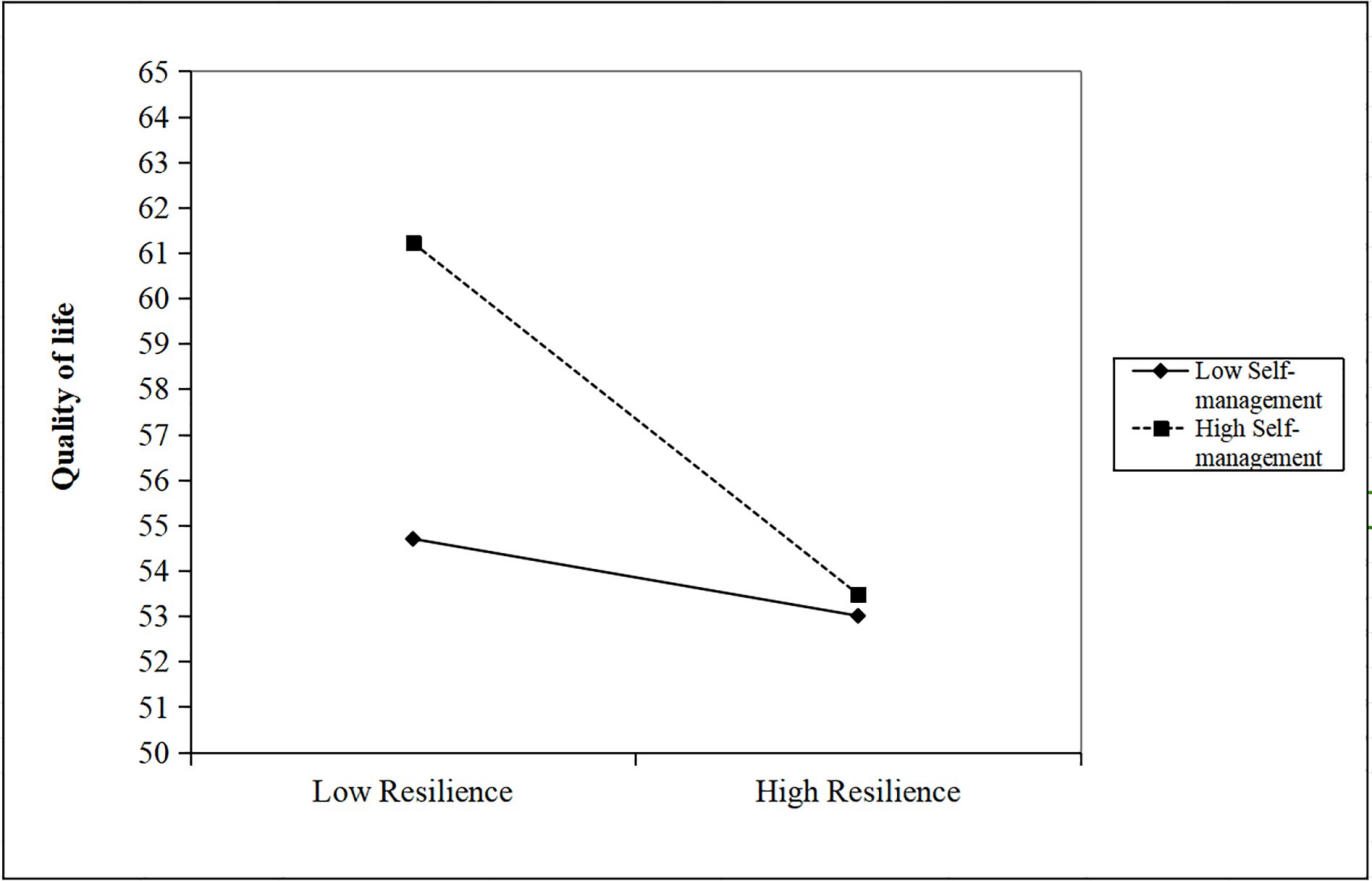
James Gaskin plot showing the interaction effect of resilience and self-management on quality of life.

Further analysis, using a simple slope test, clarified these interactions. The test results, depicted in Fig 4, indicate a significantly stronger negative relationship between self-efficacy and QOL scores at higher levels of self-management ***(simple slope = -2*.*733*, *p < 0*.*0001)***. Similarly, as illustrated in Fig 5, for individuals with elevated levels of self-management, there was a significantly enhanced negative association between resilience and QOL scores ***(simple slope = -0*.*252*, *z < 0*.*0001)***. Collectively, these results underscore the fact that enhanced self-management capabilities significantly bolster the positive effects of self-efficacy and resilience on QOL.

## Discussion

This is the first study to explore the mediating role of resilience and moderating role of self-management on QOL among patients with T2DM. The results of this study confirmed that the effect of self-efficacy on QOL is mediated, in part, by resilience, which provides useful information for designing precise interventions to improve QOL in patients with T2DM. Moderation analysis showed that resilience and self-efficacy significantly affected QOL with high or low self-management behaviors in patients with T2DM. This study confirmed Hypothesis 1, which stated that resilience mediates the relationship between self-efficacy and QOL. The effect of self-efficacy on the QOL of patients with diabetes can be understood through both direct and indirect pathways. SEM demonstrated a substantial direct negative relationship between self-efficacy and QOL scores (β = -0.753, *p* < 0.001). These results underscore the association between elevated self-efficacy and improved QOL in individuals with T2DM. This aligns with other studies showing that self-efficacy is a predictor of QOL in patients with chronic diseases and is a key determinant of encouraging positive actions [30]. Another study showed that high self-efficacy was significantly correlated with good glycemic control and well-being in patients with T2DM [31]. According to the information-motivation-behavioral skills model theory, people are more likely to take health-related actions, such as DSM behaviors, if they are well informed, highly motivated, and have adequate and appropriate behaviors that lead to positive health outcomes, which are essential for improving QOL [32]. In this context, diabetic self-efficacy can be understood as a behavioral skill for improving DSM behaviors. Patients with higher self-efficacy exhibit more positive disease perceptions, better adaptability, and greater confidence in disease management, which are essential for enhancing QOL [33]. Such patients experience lower disease uncertainty, effectively utilize social resources, adopt efficient coping strategies, and promote benefit discovery, thereby improving their QOL. Previous findings have shown that subjective well-being, defined as an individual’s overall evaluation of life quality according to personal standards, is higher in patients with elevated self-efficacy owing to more positive emotions and life assessments [34], the higher the patient’s rating of their overall QOL.

The SEM analysis results also confirmed that self-efficacy significantly predicted resilience in patients with T2DM (β = 3.442, *p* < 0.001). In other words, higher self-efficacy scores correlated with increased resilience and a stronger ability to withstand stress. An intermediary pathway existed between self-efficacy and QOL, with the intermediary effect accounting for 43.1% of the total effect. This suggests that patients with T2DM with high self-efficacy levels exhibit greater resilience and, ultimately, better QOL. This finding is consistent with that of previous studies that patients with diabetic foot ulcers and high psychological resilience had significantly higher self-efficacy, general health, vitality, social functioning, emotional roles, and mental health than those with low psychological resilience [35]. Self-efficacy is defined as an individual’s perceived capacity to execute particular tasks, essentially their self-confidence, which influences the effort exerted in activities and the persistence and resilience shown during setbacks [36]. Good psychological resilience allows individuals to mobilize protective factors to overcome difficulties and setbacks, leading to more successful experiences and higher self-efficacy. An earlier study defined diabetes resilience as the achievement of one or more positive diabetes outcomes such as self-perceived good QOL and glycemic outcomes within target ranges, despite the challenges associated with T2DM [37]. The compensation model of resilience [38] posits that resilience can offset the influence of stressors or adverse events, helping individuals maintain internal mental stability. Another study showed that positive emotion regulation strategies can enhance resilience, whereas emotional regulation self-efficacy and internal control sources of emotional experience significantly predict mental toughness [39]. According to Lazarus’ stress and coping theory [40], stress arises when environmental or internal demands exceed available resources. The impact of stressors primarily depends on cognitive evaluation and coping strategies. As a perceived coping resource, self-efficacy influences patients’ cognitive evaluations of stressors and mental health outcomes. Patients with high self-efficacy possess more tenacious inner qualities, can establish a positive cognitive evaluation system, correctly address their diseases, actively cope with adverse reactions, and reduce anxiety and depression, thereby enhancing their confidence and ability to manage stress, resulting in higher psychological resilience [41]. Numerous studies have demonstrated that resilience is a significant factor in post-traumatic growth [42,43]. Patients with high resilience and strong self-emotion regulation abilities adopt positive emotion regulation and effective coping strategies for adversity, enhancing resilience and toughness, rebuilding confidence, and fostering positive emotions. Consequently, post-traumatic growth reflects these positive changes. Individuals with elevated levels of resilience tend to exhibit a greater propensity to initiate constructive transformations and improve their QOL after traumatic events [44]. Consequently, self-efficacy and resilience are essential internal positive psychological traits pivotal in maintaining and enhancing QOL, psychological health, and personality development [14]. Healthcare professionals can conduct resilience-promoting interventions [45] and patient-centered care models based on self-efficacy [46] or information-motivation-behavioral skills intervention programs based on protection motivation theory [47], which aim to enhance resilience, improve self-efficacy, cultivate positive cognition, and emotional management abilities, encourage optimism, generate psychological security, and improve QOL.

The findings for Hypothesis 2 suggest that self-management behaviors moderate the relationship between self-efficacy and QOL. This aligns with previous research, where a meta-analysis has demonstrated the positive impact of self-management programs on the QOL of patients with prostate cancer and enhancement of their self-efficacy [48]. In essence, patients with T2DM with better disease control exhibit superior self-management, higher self-identity, elevated self-efficacy, and improved QOL. Effective long-term disease management in patients with T2DM requires a significant level of self-efficacy [49]. In light of Bandura’s self-efficacy theory [16], the realization of the influence of self-efficacy on QOL is fundamentally linked to behavior. Self-efficacy primarily functions by regulating behaviors and influencing outcomes through behavioral control. Consequently, in the context of T2DM, self-efficacy is manifested through the modulation of self-management behaviors. Patients with T2DM are more inclined to complete tasks or behaviors that they feel competent in handling, with successful behavioral experiences providing positive feedback that enhances self-efficacy. Positive reinforcement of successful self-management behaviors further boosts self-efficacy through positive feedback. Protective motivation can enhance self-management awareness and action by stimulating self-protective behaviors and improving disease perception and control [50]. It promotes better disease control, reduces glucose levels, and improves QOL. Patients with a high level of self-management have effective problem solving and self-care abilities that help identify and manage complications, while positive emotions and supportive partnerships enhance patients’ confidence and determination to overcome the disease, leading to better disease control, reduced medical costs, and improved QOL. Another study indicated that a management platform based on the Capacity, Opportunity, Motivation-Behavior model could improve patients’ self-management abilities and self-efficacy in diabetes care [51].

We also found that self-management behaviors moderated the second half of the mediating pathway through which self-efficacy affected QOL through resilience. This finding indicates that effective self-management amplifies the positive impact of resilience on QOL. This is consistent with the results of a previous study that showed that diabetes-related self-care activities are associated with positive health [52]. High levels of self-management facilitate better adaptation to changes in life, making positive outcomes more attainable after experiencing stress and adversity. Patients who embrace a long-term self-management model, believe in their pressure-resistance capabilities, and accumulate strength exhibiting higher resilience. A study shows that promoting diabetes management self-efficacy can be an effective strategy for enhancing resilience and decreasing diabetes distress [17]. According to the process integration model of resilience, mental toughness is a dynamic process in which individuals interact with psychologically protective factors during adverse events [53]. Patients with high self-management skills confidently and positively utilize various social support resources to address disease challenges. Greater mental toughness is correlated with proactive health behaviors that alleviate disease-related pain and negative emotions, leading to comprehensive disease management [54]. Achieving lifestyle changes and glucose control goals fosters a robust cognitive evaluation system that allows patients to strategize behaviors, adjust disease perceptions, and continually optimize actions [55]. This approach helps patients cope with difficulties, reduces anxiety and pain, and improves resilience. Successful self-management experiences provide positive feedback, enhance self-efficacy, and mediate the effect of resilience. Psychosocial factors play a central role in behavioral changes and self-management. Self-management facilitates self-efficacy and resilience. Effective self-management may lead to better outcomes such as glucose control, health status, and QOL [56]. Healthcare providers should address these issues to resolve significant self-management problems. These strategies include employing structured education rooted in the protection motivation theory [57] and utilizing continuous care through Internet-based platforms [58]. Encouraging patients to acquire self-management skills and diabetes practice training enhances cognitive and psychological self-management, including emotional adjustment, cognitive reconstruction, and maintenance of a positive attitude, thus significantly improving QOL from the perspective of positive psychology.

### Limitations

This study’s contributions are tempered by several limitations that warrant caution when interpreting its findings. The use of a cross-sectional design permits only the identification of associations among the studied variables and not causal linkages. Consequently, future research employing longitudinal or experimental designs is crucial for establishing causality. The analysis examined self-efficacy, resilience, and self-management as the primary influencers. Subsequent research should be extended to include other positive psychological and physiological factors that may enhance the QOL of patients with T2DM. Finally, the recruitment of participants from a single medical center may have affected the generalizability of the results to broader populations.

### Implications

To the best of our knowledge, this is the first study to explore the direct and mediated roles of self-efficacy, resilience, and self-management in influencing the QOL of individuals with T2DM. This study has several critical implications for clinical practice. First, healthcare providers should focus on simultaneously enhancing self-efficacy and resilience to reduce diabetes-related distress, which is crucial for maintaining positive psychological well-being in patients with T2DM, thereby potentially improving their QOL. Second, optimal self-management levels modulate the impact of self-efficacy and resilience on QOL. Healthcare professionals should encourage patients to develop beneficial health behaviors and utilize effective strategies to augment their self-efficacy and resilience, which can significantly improve their QOL. Third, it is imperative for healthcare providers to consider the role of positive psychological factors and continuously seek methods to enhance self-management practices as these components are instrumental in improving the overall QOL of patients with T2DM.

## Conclusion

The moderated mediation model proposed in this study elucidates the mechanisms and pathways that influence the QOL of patients with T2DM from a positive psychology standpoint. The results validate that the link between self-efficacy and QOL is mediated by resilience, while self-management acts as a moderator. Thus, augmenting self-efficacy and resilience can significantly enhance QOL, and the promotion of self-management behaviors serves as another pivotal strategy for these improvements in patients with T2DM. It is essential for nurses and other healthcare practitioners to pay specific attention to helping patients have a more positive mindset, by devising specialized interventions to boost their self-management capabilities and, consequently, their QOL.

## Supporting information

S1 Table Chinese version questionnaire. (DOCX)

S1 File Compressed/ZIP file archive. (ZIP)
