# Supplementary material for: Exploring the dynamics of self-efficacy, resilience, and self-management on quality of life in type 2 diabetes patients: A moderated mediation approach from a positive psychology perspective
Source: PLoS One. 2025 Jan 24;20(1):e0317753. doi: 10.1371/journal.pone.0317753 (PMC11759368; doi:10.1371/journal.pone.0317753)
Supplement: S1 Table — (DOCX) [file pone.0317753.s001.docx]

**Table S1: Chinese version questionnaire**

**表1**

**(一)请您根据您的实际情况，在“＿＿”上填写内容，或在适当的选项上打“√”**。

1. **性别：**(1)男 (2)女
2. **年龄：＿＿岁**
3. **文化程度：**⑴未接受正规学校教育 ⑵小学 ⑶初中 ⑷高中/中专/技校 ⑸大专 ⑹本科 ⑺研究生及以上
4. **婚姻状况：**(1)未婚 (2)已婚 (3)离异 (4)丧偶 (5)分居
5. **家庭人均月收入(元)：**(1)＜3000 (2)3000-5000 (3)5000-10000 (4)10000以上
6. **医疗费用支付方式：**

(1)城镇职工基本医疗保险 (2)农村合作医疗保险 (3)商业保险 (4)自费

1. **家庭住址：**(1)农村 (2)乡镇 (3)市区
2. **身高：**＿＿＿cm **体重：**＿＿＿kg
3. **有无糖尿病家族史：**(1)没有 (2)有（父亲/母亲/兄弟姐妹）
4. **糖尿病确诊时间：**＿＿年＿＿月
5. **有无并发症：**(1)没有 (2)有（可多选）①视网膜病变②肾病③周围神经病变 ④周围血管病变 ⑤糖尿病足⑥糖尿病皮肤病
6. **有无合并症：**(1)没有 (2)有（可多选）①心脑血管疾病②高血脂③关节炎 ④呼吸系统疾病⑤高尿酸血症⑥肝脏疾病⑦耳朵/鼻子/喉咙问题 ⑧高血压 ⑨其他＿＿＿
7. **目前治疗方式：**(1)不使用降糖药 (2)口服降糖药（磺脲类/双胍类/α糖苷酶抑制剂/其他） (3)使用胰岛素（速效/短效/中效/长效/预混） (4)口服降糖药+胰岛素
8. **是否主动获取糖尿病居家治疗、自我管理相关知识：**(1)是(2)否 (获取途径为 ：网络、书籍、健康教育课堂、医务人员讲解等。)

**表2：请您仔细阅读下面每个阐述，根据您当下的主观感受填写，谢谢！**

| **序号** | **项目** | **0天** | **1天** | **2天** | **3天** | **4天** | **5天** | **6天** | **7天** |
| --- | --- | --- | --- | --- | --- | --- | --- | --- | --- |
| 1 | 在过去7天中，您有多少天 ，按照健康的饮食计划来进食？ |  |  |  |  |  |  |  |  |
| 2 | 近1个月您平均每周有多少天按糖尿病饮食要求合理安排饮食？ |  |  |  |  |  |  |  |  |
| 3 | 在过去7天中，您有多少天，一天所持的蔬菜水果加起来超过5份，如：吃蔬菜3碟、水果2个(水果一份约橘子一个，木瓜1/3个...等；蔬菜一份100克，约1碟）？ |  |  |  |  |  |  |  |  |
| 4 | 在过去7天中，您有多少天吃油脂多的食物（如：油炸食物、肥肉、鸡皮等）？ |  |  |  |  |  |  |  |  |
| 5 | 在过去7天中，您有多少天，有做30分钟以上的活动（指身体持续活动超过30分钟，包括：走路、做家务）？ |  |  |  |  |  |  |  |  |
| 6 | 在过去7天，除了工作及做家务事以外，您有多少天，有另外拨时间去做运动（如：慢跑、爬山、太极拳等）？ |  |  |  |  |  |  |  |  |
| 7 | 在过去7天中，您有多少天，在家自己（或家人帮忙）量血糖？ |  |  |  |  |  |  |  |  |
| 8 | 在过去7天中，您有多少天，依照医师指示的血糖测量标准次数（例如1天量两次）在家按时自己（或家人帮忙）量血糖？ |  |  |  |  |  |  |  |  |
| 9 | 在过去7天中，您有多少天，检查您的双脚（包括脚趾、脚板与脚底）？ |  |  |  |  |  |  |  |  |
| 10 | 在过去7天中，您有多少天，在穿鞋之前有先检查鞋内情形（如：鞋内有无小石头、是否平整、有无破损或潮湿）？ |  |  |  |  |  |  |  |  |
| 11 | 在过去7天中，您有多少天，有照医师指示定时定量服用降糖的药或注射胰岛素？ |  |  |  |  |  |  |  |  |

**表3：请您仔细阅读下面每个阐述，根据您当下的主观感受填写，谢谢！**

1. **您认为糖尿病对您的健康损害有多大?**

(1)根本没损害 (2)有点 (3)中度 (4)重度 (5)极重度

1. **您经常有皮肤瘙痒，肢体麻木,疼痛等身体不舒适的感觉吗?**

(1)根本没有 (2)偶尔有 (3)有（约一半时间） (4)经常有 (5)总是有

1. **身体不舒适的感觉对您的生活有多大干扰?**

(1)根本没干扰 (2)有点干扰 (3)有干扰（中度） (4)很干扰 (5)极大干扰

1. **您是否感觉看东西越来越困难?**

(1)根本没有 (2)偶尔有 (3)有（约一半时间） (4)经常有 (5)总是有

1. **视力的下降对您的日常生活有多大影响?**

(1)根本没有 (2)有点影响 (3)有影响（中度） (4)很大影响 (5)极大影响

1. **您是否感觉听清别人讲话越来越困难?**

(1)根本没有 (2)偶尔有 (3)有（约一半时间） (4)经常有 (5)总是有

1. **听力的下降对您的日常生活有多大的影响?**

(1)根本没有 (2)有点影响 (3)有影响（中度） (4)很大影响 (5)极大影响

1. **您是否常感到胸痛,胸闷和心悸?**

(1)根本没有 (2)偶尔有 (3)有（约一半时间） (4)经常有 (5)总是有

1. **您是否感到皮肤和脚很容易感染?**

(1)根本没有 (2)偶尔有 (3)有（约一半时间） (4)经常有 (5)总是有

1. **皮肤和脚的感染对您的生活有多大影响?**

(1)根本没有 (2)有点影响 (3)有影响（中度） (4)很大影响 (5)极大影响

1. **您是否觉得对外界事物的反应能力下降了?**

(1)根本没有 (2)有点 (3)下降了（中度） (4)下降很大 (5)下降极大

1. **您是否总感觉饥饿?**

(1)根本没有 (2)偶尔有 (3)有（约一半时间） (4)经常有 (5)总是有

1. **糖尿病经常给您的日常生活带来麻烦和不便了吗?**

(1)根本没有 (2)偶尔有 (3)有（约一半时间） (4)经常有 (5)总是有

1. **您是否经常想糖尿病对您意味着什么?**

(1)根本没有 (2)偶尔有 (3)有（约一半时间） (4)经常有 (5)总是有

1. **您是否担忧您会突然死掉**

(1)根本不 (2)偶尔担忧 (3)有（约一半时间） (4)经常担忧

(5)总是担忧

1. **饮食控制是否使您感到烦恼?**

(1)根本没烦恼 (2)偶尔烦恼 (3)烦恼(约一半时间) (4)经常烦恼

(5)总是烦恼

1. **定期自测尿糖或到医院检查血糖使您感到麻烦吗?**

(1)根本没有 (2)偶尔有 (3)有（约一半时间） (4)经常有 (5)总是有

1. **您是否因糖尿病而感到紧张或局促不安?**

(1)根本没有 (2)偶尔有 (3)有（约一半时间） (4)经常有 (5)总是有

1. **您对您目前的治疗效果满意吗?**

(1)极满意 (2)很满意 (3)满意（中度） (4)很不满意 (5)极不满意

1. **您是否相信您能战胜疾病的困扰?**

(1)根本不 (2)有点相信 (3)相信（中度） (4)很相信 (5)极相信

1. **您认为糖尿病对您的人际关系是否损害?**

(1)根本没有 (2)有点损害 (3)中度损害 (4)很损害 (5)极度损害

1. **您是否感到因为患有糖尿病而被人嫌弃?**

(1)根本没有 (2)偶尔有 (3)有(约一半时间) (4)经常有 (5)总是有

1. **糖尿病对您在家里或单位里的地位和作用有影响吗?**

(1)根本没有 (2)有点影响 (3)有影响(中度) (4)很大影响 (5)极大影响

1. **您经常和周围的病友交流有关糖尿病的体验,问题和知识吗？**

(1)根本不交流 (2)偶尔有 (3)有(约一半时间) (4)经常有 (5)总是有

1. **您服药后是否有过敏、恶心等药物不良反应？**

(1)根本没有 (2)偶尔有 (3)有（约一半时间） (4)经常有 (5)总是有

1. **您是否有心悸、头昏和出虚汗等低血糖反应吗？**

(1)根本没有 (2)偶尔有 (3)有（约一半时间） (4)经常有 (5)总是有

1. **饮食控制对您的生活方式或生活习惯有多大限制？**

(1)根本没有 (2)有点限制 (3)有限制（中度） (4)很受限制 (5)极受限制

**表4：请您仔细阅读下面每个阐述，根据您当下的主观感受填写，谢谢！**

| 序号 | 题目 | 从来不0 | 很少  1 | 有时  2 | 经常  3 | 一直如此4 |
| --- | --- | --- | --- | --- | --- | --- |
| 1 | 我能适应变化 |  |  |  |  |  |
| 2 | 我有亲密、安全的关系 |  |  |  |  |  |
| 3 | 有时，命运或上帝能帮忙 |  |  |  |  |  |
| 4 | 无论发生什么我都能应付 |  |  |  |  |  |
| 5 | 过去的成功让我有信心面对挑战 |  |  |  |  |  |
| 6 | 我能看到事情幽默的一面 |  |  |  |  |  |
| 7 | 应对压力使我感到有自强 |  |  |  |  |  |
| 8 | 经历艰难或疾病后，我往往会很快恢复 |  |  |  |  |  |
| 9 | 事情发生总是有原因的 |  |  |  |  |  |
| 10 | 无论结果怎样，我都会尽自己最大努力 |  |  |  |  |  |
| 11 | 我能实现自己的目标 |  |  |  |  |  |
| 12 | 当事情看起来没什么希望时，我不会轻易放弃 |  |  |  |  |  |
| 13 | 我知道去哪里寻求帮助 |  |  |  |  |  |
| 14 | 在压力下，我能够集中注意力并清晰思考 |  |  |  |  |  |
| 15 | 我喜欢在解决问题时起带头作用 |  |  |  |  |  |
| 16 | 我不会因失败而气馁 |  |  |  |  |  |
| 17 | 我认为自己是个强有力的人 |  |  |  |  |  |
| 18 | 我能做出不寻常的或艰难的决定 |  |  |  |  |  |
| 19 | 我能处理不快乐的情绪 |  |  |  |  |  |
| 20 | 我不得不按照预感行事 |  |  |  |  |  |
| 21 | 我有强烈的目的感 |  |  |  |  |  |
| 22 | 我感觉能掌控自己的生活 |  |  |  |  |  |
| 23 | 我喜欢挑战 |  |  |  |  |  |
| 24 | 我努力工作以达到目标 |  |  |  |  |  |
| 25 | 我对自己的成绩感到骄傲 |  |  |  |  |  |

**表5：请您仔细阅读下面每个阐述，根据您当下的主观感受填写，谢谢！**

| 序号 | 题目 | 完全没有信心-------------------------完全有信心 | | | | | | | | | |
| --- | --- | --- | --- | --- | --- | --- | --- | --- | --- | --- | --- |
| 1 | 您有信心做到每天早、中、晚三餐都在固定的时间、以固定的饭量吃饭吗？ | 1 | 2 | 3 | 4 | 5 | 6 | 7 | 8 | 9 | 10 |
| 2 | 您在跟非糖尿病患者同时进餐时，对仍坚持自己的饮食的量和种类（原来吃什么还是吃什么，原来吃多少，还是吃多少），您有信心做到吗？ | 1 | 2 | 3 | 4 | 5 | 6 | 7 | 8 | 9 | 10 |
| 3 | 当您饥饿时，您有信心挑选出合适的食物（如零食等）吗？ | 1 | 2 | 3 | 4 | 5 | 6 | 7 | 8 | 9 | 10 |
| 4 | 对于每次运动15-30分钟，每周4-5次，您有多大信心做到？ | 1 | 2 | 3 | 4 | 5 | 6 | 7 | 8 | 9 | 10 |
| 5 | 在运动时，您有信心避免低血糖的发生吗？ | 1 | 2 | 3 | 4 | 5 | 6 | 7 | 8 | 9 | 10 |
| 6 | 当您的血糖升高或降低时，您对于做出正确的处理有信心吗？ | 1 | 2 | 3 | 4 | 5 | 6 | 7 | 8 | 9 | 10 |
| 7 | 在您的身体发生变化时，对于能够对身体状况做出判断从而及时就医您有信心吗？ | 1 | 2 | 3 | 4 | 5 | 6 | 7 | 8 | 9 | 10 |
| 8 | 对于控制您自身的糖尿病病情从而使它不影响到您的生活，您有信心做到吗？ | 1 | 2 | 3 | 4 | 5 | 6 | 7 | 8 | 9 | 10 |

再次感谢您在百忙之中填写问卷，欢迎您加入“糖尿病健康管理微信圈”，我们将会为您提供相关健康资讯及健康管理服务，请您留下联系方式，感谢您的参与与支持！

填表日期： 年 月 日
