# Supplementary material for: Exploring the dynamics of self-efficacy, resilience, and self-management on quality of life in type 2 diabetes patients: A moderated mediation approach from a positive psychology perspective
Source: PLoS One. 2025 Jan 24;20(1):e0317753. doi: 10.1371/journal.pone.0317753 (PMC11759368; doi:10.1371/journal.pone.0317753)
Supplement: S1 File — (ZIP) [file pone.0317753.s002.zip › Supporting information/S2 Data availability.docx]

**Sample characteristics**

**Table 1 Participant characteristics (N = 408)**

| Characteristic | Mean±SD | Frequency | Percentage |
| --- | --- | --- | --- |
| **Age（years）** | 57.76±10.79 |  |  |
| ＜40 |  | 32 | 7.84 |
| 41-50 |  | 62 | 15.20 |
| 51-60 |  | 141 | 34.56 |
| 61-70 |  | 127 | 31.13 |
| 71-80 |  | 46 | 11.27 |
| **Gender** |  |  |  |
| Female |  | 172 | 42.20 |
| Male |  | 236 | 57.80 |
| **Years of illness** |  |  |  |
| 0-5 |  | 98 | 24.02 |
| 5-10 |  | 97 | 23.77 |
| 11-15 |  | 94 | 23.04 |
| 16-20 |  | 66 | 16.18 |
| ＞20 |  | 53 | 12.99 |
| **BMI（kg/m^2^）** |  |  |  |
| BMI＜18.5 |  | 6 | 1.47 |
| 18.5≤BMI≤23.9 |  | 136 | 33.33 |
| 24≤BMI≤27.9 |  | 185 | 45.34 |
| BMI≥28 |  | 81 | 19.85 |
| **Education levels** |  |  |  |
| Illiterate |  | 75 | 18.38 |
| Less than high school diploma |  | 193 | 47.3 |
| High school diploma |  | 83 | 20.34 |
| Academic |  | 57 | 13.97 |
| **Marital status** |  |  |  |
| Single |  | 46 | 11.27 |
| Married |  | 362 | 88.73 |
| **Per capita monthly household income (CNY)** |  |  |  |
| ＜3000 |  | 64 | 15.69 |
| 3000-5000 |  | 176 | 43.14 |
| 5000-10000 |  | 127 | 31.13 |
| ＞10000 |  | 41 | 10.05 |
| **Payment of medical expenses** |  |  |  |
| Basic medical insurance system for urban works |  | 350 | 85.78 |
| Rural Cooperative medical insurance |  | 46 | 11.27 |
| Others |  | 12 | 2.95 |
| **Address** |  |  |  |
| Rural area |  | 75 | 18.38 |
| Urban area |  | 333 | 81.62 |
| **Family history of the disease** |  |  |  |
| No |  | 186 | 45.59 |
| Yes |  | 222 | 54.41 |
| **Complications** |  |  |  |
| No |  | 210 | 51.47 |
| 1 Complication |  | 77 | 18.87 |
| 2 Complications |  | 89 | 21.81 |
| 3 Complications |  | 25 | 6.13 |
| 4 or 5 Complications |  | 7 | 1.72 |
| **Comorbidity** |  |  |  |
| No |  | 162 | 39.71 |
| 1 Comorbidity |  | 97 | 23.77 |
| 2 Comorbidity |  | 91 | 22.30 |
| 3 Comorbidity |  | 43 | 10.54 |
| 4 or 5 Comorbidity |  | 15 | 3.68 |
| **Diabetes-Related Medication Use** |  |  | 3.68 |
| None |  | 16 | 3.92 |
| Oral Meds |  | 125 | 30.64 |
| Insulin |  | 47 | 11.52 |
| Both |  | 220 | 53.92 |
| **Actively sought out information on diabetes treatment** |  |  |  |
| Yes |  | 336 | 82.35 |
| No |  | 72 | 17.65 |

Sample characteristics: data analysis was performed using ***SPSS 26.0***, with double data entries to ensure accuracy. Qualitative data were described as ***case numbers and percentages (%),*** while quantitative data conforming to a normal distribution were summarized as ***mean ± standard deviation (x ± s)***.

| **Age** | | | |  | |
| --- | --- | --- | --- | --- | --- |
|  | N | Minimum value | Maximum value | Means | Standard deviations |
| Age(years) | 408 | 26.00 | 80.00 | 57.76 | 10.78 |

The mean age of participants in the present study was 57.76 ± 10.79 years, with 42.4% being over 60 years of age.

**Mediation analyses**

**Table 2** **Correlations, means and standards deviations of study variables**

|  | Self-efficacy | Resilience | Self-management | Quality of life |
| --- | --- | --- | --- | --- |
| Self-efficacy | 1 |  |  |  |
| Resilience | 0.347** | 1 |  |  |
| Self-management | 0.364** | 0.072 | 1 |  |
| Quality of life | -0.179** | -0.276** | 0.100* | 1 |

** *p* < 0.01, * *p* < 0.05

Variable relationships were examined using ***Spearman’s correlation analysis***. Correlation analysis revealed that self-efficacy exhibited a positive and significant association with resilience (r = 0.347, p < 0.01) and self-management (r = 0.364, p < 0.01). Furthermore, there was a significant negative correlation between self-efficacy and QOL scores (r = -0.179, p < 0.01). Resilience also demonstrated a significant negative correlation with QOL scores (r = -0.276, p < 0.01) (Table 2).

**Table 3 Total, direct, and indirect effects of each path in this model using structural equation model**

| path |  | β | SE | BC95%CI | |
| --- | --- | --- | --- | --- | --- |
|  |  |  |  | Lower | Upper |
| **Total effect** |  |  |  |  |  |
| Self-efficacy→Quality of life | | -1.534*** | 0.350 | -2.420 | -0.753 |
| **Direct effect** |  |  |  |  |  |
| Self-efficacy→Resilience | | 3.442*** | 0.403 | 2.523 | 4.414 |
| Self-efficacy→Quality of life | | -0.753* | 0.367 | -1.476 | -0.031 |
| **Indirect effect** |  |  |  |  |  |
| Self-efficacy→Resilience→Quality of life | | -0.781*** | 0.226 | -1.283 | -0.41 |

*N*=408

* p < 0.05, ** p < 0.01, *** p < 0.001

The ***structural equation modeling (SEM)*** approach was applied using ***IBM SPSS AMOS version*** 26 to explore the mediating effect of resilience on self-efficacy and QOL. Prior to data analysis, the dataset was thoroughly checked for missing values, outliers, and normal distributions. Confirmatory factor analysis was employed to validate the measurement model and assess the correlations between the observed variables and latent constructs. All factor loadings were greater than 0.50, indicating satisfactory values. The model’s goodness-of-fit was confirmed by modified indices: χ2/df = 1.646 (29.627/18 < 3), GFI = 0.984, AGFI = 0.960, CFI = 0.992, TLI = 0.984, NFI = 0.980, and RMSEA = 0.04, all suggesting a strong fit with the observed data(The model’s fit was examined utilizing several criteria: χ2/df < 5, goodness of fit index (GFI) > 0.90, Tucker-Lewis index (TLI) > 0.90, comparative fit index (CFI) > 0.90, root mean square error of approximation (RMSEA) < 0.080, and standardized root mean square residual (SRMR) < 0.080, ensuring the model adequately matched the observed data.) Bias-corrected bootstrap CI based on 5,000 samples were used to test direct and indirect effects. The absence of 0 in the CI confirmed the significance of this correlation.


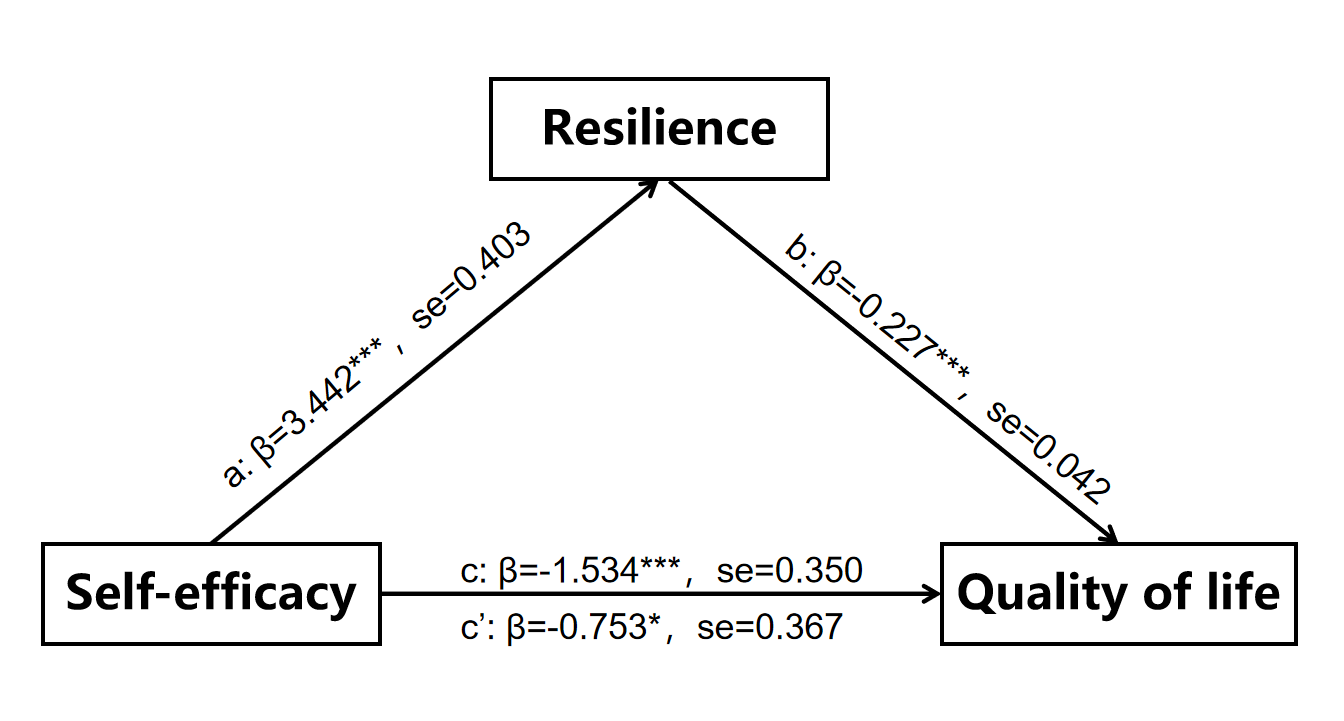


**Fig. 2** Hypothesized mediated model. Path c: total direct effect, path c’: direct effect. a: effects of Self-efficacy on the mediator (Resilience); b: effects of the mediator on the Quality of life. Using structural equation model (SEM)

As depicted in ***Table 3 and Fig 2***, the total effect of self-efficacy on QOL (path c) was significant (t = -4.381, β = -1.534, p < 0.001). The direct effect of self-efficacy on QOL (path c’) was also significant (β = -0.753, 95% CI = [-1.476, -0.030], p < 0.05). Furthermore, the indirect effect of self-efficacy on QOL, mediated by resilience, was significant (β = -0.781, 95% CI = [-1.283, -0.410], p < 0.001), indicating a partially mediating effect of resilience.

**Results of moderated mediation analysis**

**Table 4 Results of the moderated mediation model analysis**

| Variable | **Model 1** Quality of life | | | | |
| --- | --- | --- | --- | --- | --- |
|  | β | SE | t | P | 95%CI |
| Gender | 0.9282 | 1.1630 | 0.7981 | 0.4253 | -1.3584，3.2147 |
| Diabetes duration | 0.2454 | 0.4441 | 0.5527 | 0.5808 | -0.6276，1.1185 |
| Age | 0.6751 | 0.5970 | 1.1308 | 0.2588 | -0.4987，1.8489 |
| Education levels | 0.1304 | 0.4893 | 0.2665 | 0.7900 | -0.8317，1.0924 |
| self-efficacy | -1.7091 | 0.3741 | -4.5679 | 0.0000 | -2.4447，-0.9735 |
| resilience | -0.1532 | 0.0384 | -3.9860 | 0.0001 | -0.2287，-0.0776 |
| self-management | 0.1209 | 0.0450 | 2.6847 | 0.0076 | 0.0324，0.2094 |
| self-management×self-efficacy | -0.0751 | 0.0240 | -3.1330 | 0.0019 | -0.1223，-0.0280 |
| self-management×resilience | -0.0073 | 0.0029 | -2.5405 | 0.0115 | -0.0129，-0.0016 |
| R^2^ | 0.3123 | | | | |
| F | 11.0988 | | | | |

Using Hayes’ PROCESS (Model 15) in the SPSS

BMI Body mass index, CI Confidence interval

N = 408

A moderated mediation analysis was tested using ***Hayes’s PROCESS 4.1 for SPSS v. 26 (Model 15)***. This analysis included 95% bootstrap confidence intervals (CI) derived from 5,000 bootstrapped samples, with CIs excluding 0 denoting significant effects. Statistical significance was set at p < 0.05 (two-tailed). Control variables included sociodemographic and disease-related characteristics such as age, gender, education level, and duration of diabetes.


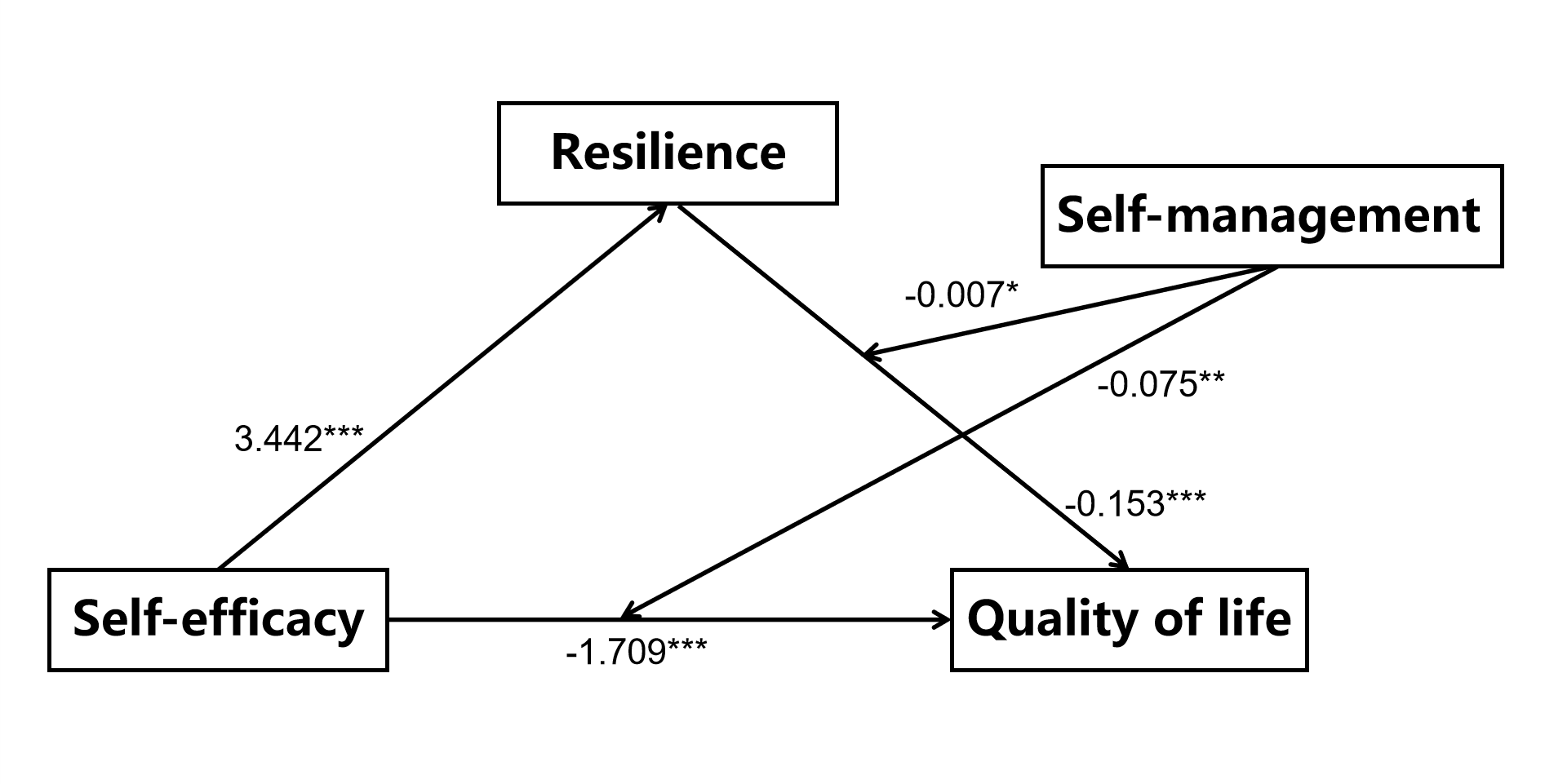


**Fig 3.** The moderated mediation model; *p < 0.05, **p < 0.01, ***p < 0.001

**Simple slope test**


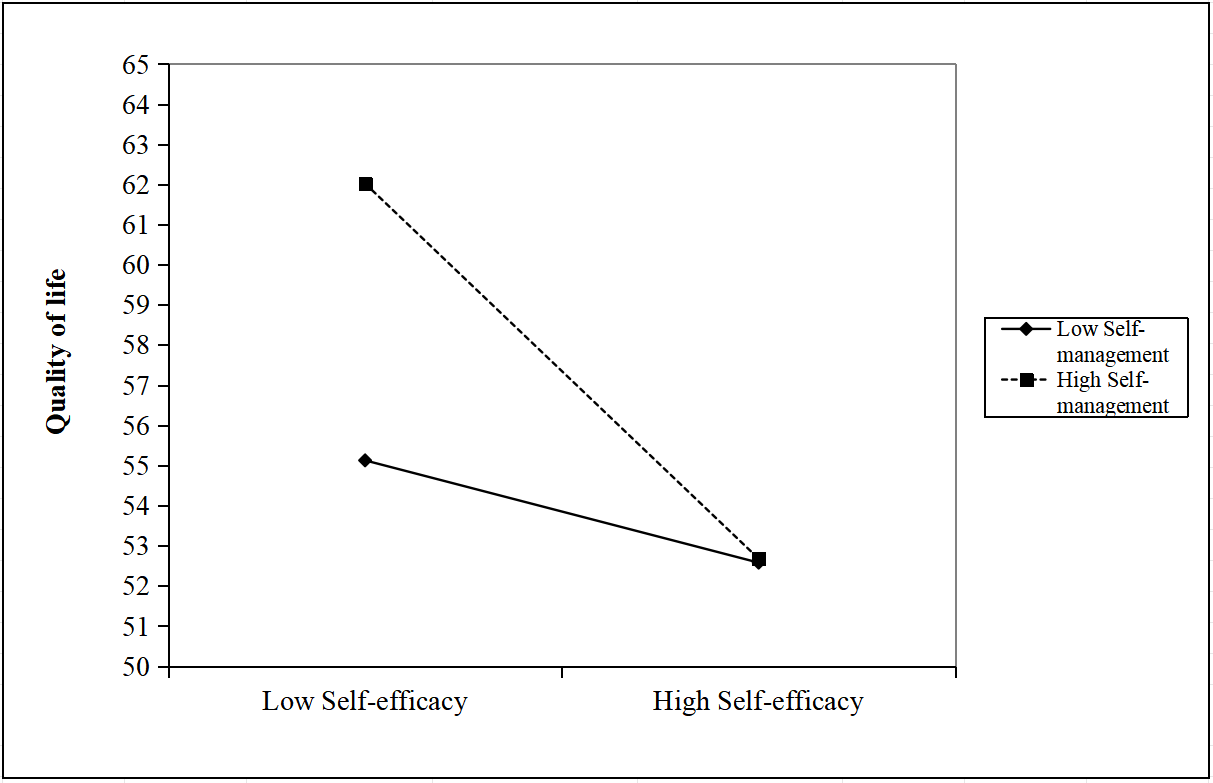


**Fig 4**. James Gaskin plot showing the interaction effect of self-efficacy and self-management on quality of life.


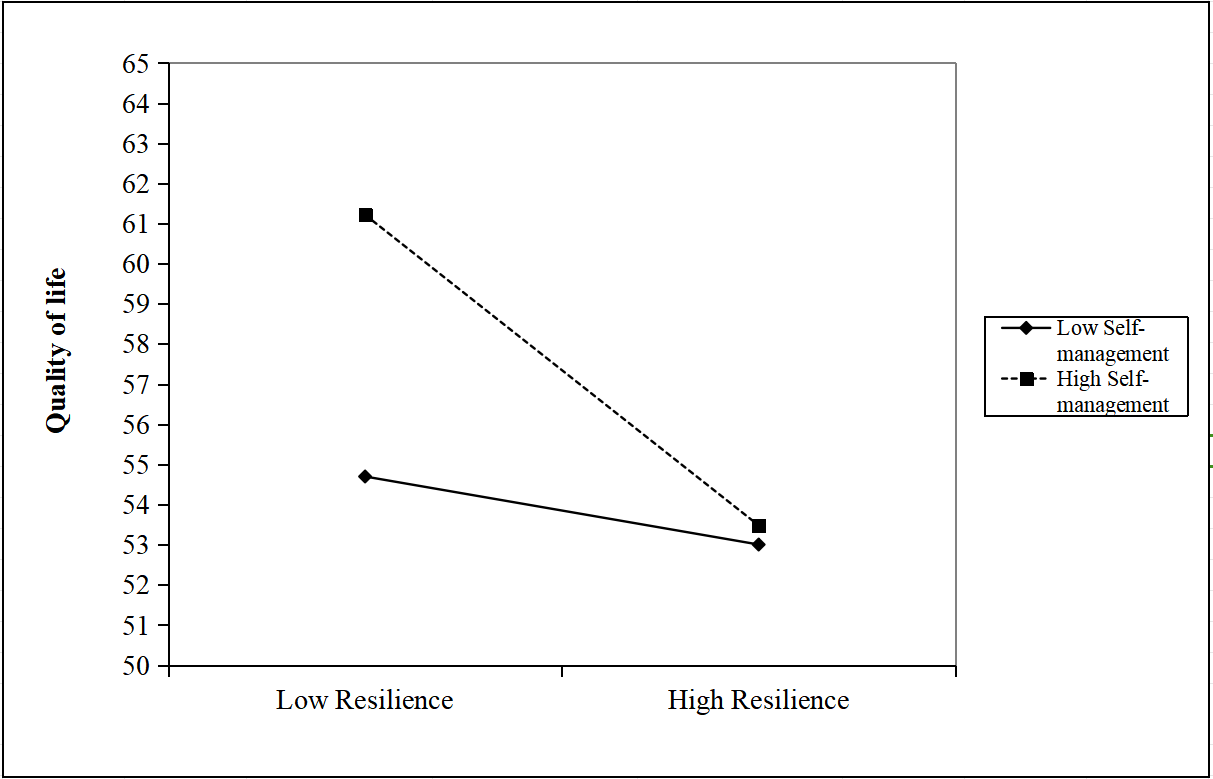


**Fig 5**. James Gaskin plot showing the interaction effect of resilience and self-management on quality of life.

Further analysis, using a ***simple slope test***, clarified these interactions. The test results, depicted in ***Fig 4***, indicate a significantly stronger negative relationship between self-efficacy and QOL scores at higher levels of self-management (simple slope = -2.733, p < 0.0001). Similarly, as illustrated in ***Fig 5***, for individuals with elevated levels of self-management, there was a significantly enhanced negative association between resilience and QOL scores (simple slope = -0.252, z < 0.0001). Collectively, these results underscore the fact that enhanced self-management capabilities significantly bolster the positive effects of self-efficacy and resilience on QOL.
